# Cycle-Inhibiting Factor Is Associated with *Burkholderia pseudomallei* Invasion in Human Neuronal Cells

**DOI:** 10.3390/biology11101439

**Published:** 2022-10-01

**Authors:** Amporn Rungruengkitkun, Niramol Jitprasutwit, Watcharamat Muangkaew, Chantira Suttikornchai, Sarunporn Tandhavanant, Nitaya Indrawattana, Sumate Ampawong, Passanesh Sukphopetch, Narisara Chantratita, Pornpan Pumirat

**Affiliations:** 1 Department of Microbiology and Immunology, Faculty of Tropical Medicine, Mahidol University, Bangkok 10400, Thailand; 2 Center for Vaccine Development, Institute of Molecular Biosciences, Mahidol University, Nakhon Pathom 73170, Thailand; 3 Department of Immunology, Faculty of Medicine Siriraj Hospital, Mahidol University, Bangkok 10700, Thailand; 4 Department of Protozoology, Faculty of Tropical Medicine, Mahidol University, Bangkok 10400, Thailand; 5 Department of Tropical Pathology, Faculty of Tropical Medicine, Mahidol University, Bangkok 10400, Thailand; 6 Mahidol-Oxford Tropical Medicine Research Unit, Faculty of Tropical Medicine, Mahidol University, Bangkok 10400, Thailand

**Keywords:** *Burkholderia pseudomallei*, cycle inhibiting factor, human neuronal cell

## Abstract

**Simple Summary:**

Neurological melioidosis, caused by *Burkholderia* *pseudomallei*, can lead to the development of severe symptoms associated with the nervous system. However, the pathogenic mechanism through which this bacterium infects neuronal cells has not been studied. This study showed that *B. pseudomallei* can infect human neuronal SH-SY5Y cells in vitro. Cycle-inhibiting factor (Cif), a type III secreted effector, is one of the virulence factors produced by *B. pseudomallei* upon infection. The *B. pseudomallei* *cif*-deleted mutant reduced the ability to invade neuronal cells compared with the parental strain. Our finding indicates that Cif is associated with *B. pseudomallei* invasion of human neuronal cells.

**Abstract:**

*Burkholderia pseudomallei* is a pathogenic bacterium that causes human melioidosis, which is associated with a high mortality rate. However, the underlying mechanisms of *B. pseudomallei* pathogenesis are largely unknown. In this study, we examined the infection of human neuronal SH-Sy5y cells by several clinically relevant *B. pseudomallei* strains. We found that all tested *B. pseudomallei* strains can invade SH-Sy5y cells, undergo intracellular replication, cause actin-tail formation, and form multinucleated giant cells. Additionally, a deletion mutant of *B. pseudomallei* cycle-inhibiting factor (*cif*) was constructed that exhibited reduced invasion in SH-Sy5y cells. Complementation of *cif* restored invasion of the *B. pseudomallei* *cif*-deleted mutant. Our findings enhance understanding of *B. pseudomallei* pathogenicity in terms of the virulence factor Cif and demonstrate the function of Cif in neurological melioidosis. This may eventually lead to the discovery of novel targets for treatment and a strategy to control the disease.

## 1. Introduction

Melioidosis is an infectious disease caused by *B. pseudomallei*, a gram-negative facultative intracellular bacillus. Melioidosis is endemic in Northern Australia and Southeast Asia, including in Myanmar, Malaysia, Singapore, Vietnam, Cambodia, Laos, and Thailand [1,2]. Furthermore, the predicted global distribution of melioidosis indicates that it is probably endemic in countries that have never reported the disease [3]. The worldwide incidence of melioidosis is approximately 165,000 cases per year, whilst the annual death rate is estimated to be approximately 89,000 [3]. Melioidosis is acquired by inhalation, inoculation, or ingestion of *B. pseudomallei* [4,5,6]. *B. pseudomallei* is a natural inhabitant of soil, stagnant water, and rice paddies where the disease is endemic. This bacterium is a category B bioterrorism agent due to its infection potential and high virulence [7]. The most common clinical symptoms are pneumonia (51%), genitourinary symptoms (14%), skin lesion (13%), and central nervous system (CNS) or neurological melioidosis (5%) [8,9].

Although neurological melioidosis is a rare condition, it is often fatal, with a mortality rate of approximately 25% [9]. Neurological abnormalities of melioidosis generally present with brain abscesses and encephalitis [10]. Symptoms of meningoencephalitis involving the brainstem, cerebellum, and spinal cord are also observed [9]. Although patients can recover entirely, 13% of patients suffer chronic neurological disability [11]. Most neurological melioidosis cases have been described in Australia [9]. In Thailand, 3% of melioidosis cases were neurological melioidosis [12]. Sporadic cases of neurological melioidosis are also reported in Norway, Taiwan, and Singapore [13,14,15]. These data show that neurological melioidosis and CNS damage caused by *B. pseudomallei* is a serious concern.

Upon infection, an essential feature of pathogenic *B. pseudomallei* is its ability to invade several cell types and to stimulate various host-cell responses [16,17]. After internalization, *B. pseudomallei* can escape the membrane-bound phagosome to enter the cytoplasm [18]. Once inside cytosol, this pathogen has evolved the ability to exploit host actin, harness actin-based motility for intra- and inter-cellular movement, and to induce cell-to-cell fusion, resulting in multinucleated giant cell (MNGC) formation [19]. This phenotype is critical for evading host defense mechanisms, including antimicrobial agents and immune response events [20]. This unique ability is also observed in the tissues of patients with melioidosis [21].

Several virulence factors of *B. pseudomallei* that contribute to pathogenesis have been identified, including both cell-associated and secreted products. Many Gram-negative pathogens, including *B. pseudomallei*, also deploy type III secretion systems (T3SSs) [22]. The T3SSs are molecular syringes/needles that inject bacterial virulence proteins directly into host cells. These injected effectors subvert host cell processes and contribute to disease [23]. In this study, we investigated a T3SS translocated effector molecule that inhibits host cell cycle progression, which is called the cycle-inhibiting factor (Cif). Enteropathogenic and enterohaemorrhagic *Escherichia coli* (EPEC and EHEC) exploit this protein to block cell cycle G2/M transition, induce stress fiber formation, and provoke a delayed cell death [24,25].

In *B. pseudomallei*, this virulence factor, which is also known as CHBP (Cif homolog in *B. pseudomallei*), is absent from genomes of closely related *B. thailandensis*, which usually do not cause human melioidosis [26]. The determination of the crystal structure showed that *B. pseudomallei* Cif possess a papain-like fold with a Cys-His-Gln catalytic triad similar to EPEC Cif [27,28]. An analysis of the T3SSs revealed that the *Burkholderia* secretion apparatus (Bsa) T3SS is required for the secretion and delivery of Cif into the host cells [29]. This effector is expressed only in intra-host cell conditions and is detected in *B. pseudomallei*-infected cells [29]. This is consistent with previous reports that *B. pseudomallei* Cif is injected into eukaryotic cells by the T3SS of EPEC and EHEC [26,27,28]. In addition, a study of the *B. pseudomallei* protein microarray probed with melioidosis patient sera identified 170 reactive antigens, including Cif, indicating that Cif is expressed in vivo and might be involved in *B. pseudomallei* pathogenesis [30].

The modus operandi of Cif is known to deamidate Nedd8, an ubiquitin-like protein, which causes the inhibition of Cullin E3 ubiquitin ligases (CRL), and consequently induces cell cycle arrest [31,32,33]. *B. pseudomallei* Cif can function as a potent activator of MAPK/ERK signaling and has CRL independent effects to counter the pro-apoptotic effects [34]. However, whether Cif employs additional roles on host cells that are crucial for bacterial pathogenesis is currently poorly understood. One study reported that Cif exerts a bimodal effect on host NF-κB signaling and bacterial replication [35]. The authors demonstrated that HEK293T cells transiently transfected with Cif increase *B. thailandensis* intracellular bacterial load, compared with the control cells transfected with the empty vector [35]. This could be linked to the consequence of disrupting the *B. pseudomallei cif* gene, causing a significant reduction in cytotoxicity and plaque formation in HeLa human epithelial cells infected by *B. pseudomallei*, as demonstrated in our previous study [29]. Therefore, Cif is likely to be important during *B. pseudomallei* infection.

To better understand the pathogenesis of *B. pseudomallei* during neurological infection, we used a cell-based model. We used the human neuroblastoma SH-SY5Y cell line to examine the pathogenic ability of three clinical wild-type *B. pseudomallei* strains, including the reference strain K96243, isolated from patients presenting with melioidosis in northeast Thailand. Furthermore, we postulated that Cif facilitates *B. pseudomallei* pathogenesis of human neuronal cells; therefore, we compared the parental strain with a *cif*-deleted mutant in SH-SY5Y cells, focusing on invasion, intracellular replication, actin-tail formation, and MNGC formation. As a result, we showed that Cif contributes to *B. pseudomallei* invasion of neuronal cells. Our in vitro model can be used to investigate the impact of other bacterial factors of virulence that contribute to the pathogenesis of neurological melioidosis by *B. pseudomallei*.

## 2. Materials and Methods

### 2.1. Ethics Statement

All experiments and methods were performed per relevant guidelines and regulations. This project has been approved by the ethics committee of the Faculty of Tropical Medicine, Mahidol University, Bangkok, Thailand (Reference No: MUTM 2018–057-01).

### 2.2. Bacterial Strains, Cell Lines, and Growth Conditions

Three *B. pseudomallei* clinical isolates (576, 1530, and the reference strain K96243), which were obtained from three patients presenting with melioidosis to Sappasithiprasong Hospital, northeastern Thailand, were used as the wild-type strains [36]. Two *B. pseudomallei* mutant strains (*cif*-deletion mutant and complemented strains) were constructed in this study as described below. *Escherichia coli* strain DH5α and RHO3 were used for cloning and generation of *B. pseudomallei* mutant strains. All *B. pseudomallei* strains and *E. coli* DH5α were grown in Luria-Bertani (LB) medium at 37 °C. *E. coli* RHO3 was grown on LB agar supplemented with 400 μg/mL 2,6-diaminopimelic acid (DAP).

The human neuronal cell line, SH-SY5Y (ATCC^®^ CRL-2266™), was kindly provided by Natthanej Luplertlop [37]. SH-SY5Y cells were maintained at 37 °C in a humidity-controlled incubator with 5% CO_2_ in Dulbecco’s modified Eagle’s medium (DMEM; Gibco BRL) supplemented with 10% (*v/v*) heat-inactivated fetal bovine serum (FBS; Gibco BRL) and penicillin-streptomycin solution (Gibco BRL). The cell culture medium was replaced with fresh medium every other day. When the cells reached approximately 90% confluence, a 0.25% (*w/v*) trypsin-EDTA solution was added after washing with PBS to harvest the cells for passaging.

### 2.3. Construction of B. pseudomallei Cif-Deleted Mutant and the Complemented Strain

The markerless allele replacement method by pEKM5 suicide vector [38] was used for deletion mutagenesis and complementation of the *cif* gene. A sequence of *B. pseudomallei* K96243 *cif* gene from GenBank (locus_tag = “BPSS1385”) was used in primer design by Primer-BLAST “http://www.ncbi.nlm.nih.gov/tools/primer-blast (accessed on 21 October 2020)”. Primer information is provided in Table 1. To generate the deletion mutant, the 5′ upstream and 3′ downstream fragments of the *cif* gene were amplified and subjected to overlap extension PCR using BPSS1385-F1 and BPSS1385-R2. The length of PCR amplicon with a deletion in the region on *cif* was 1053 bp. This overlapped PCR was ligated into pGEM^®^-T Easy (Promega, Madison, WI, USA) and then transformed into *E. coli* DH5α. The desired plasmid was validated by PCR. Additionally, the amplified PCR product was gel extracted and the deletion of *cif* was validated by DNA sequencing. The scarless knockout cassette containing a deletion in the *cif* gene was sub-cloned into the non-replicative plasmid, pEXKm5 [38], and transformed into *E. coli* RHO3 and delivered to the host *B. pseudomallei* K96243 by conjugation. LB agar containing 1000 µg/mL kanamycin and supplemented with 5-bromo-4-chloro-3-indolyl-β-D-glucuronide (X-Gluc) at a final concentration of 50 µg/mL (Promega) were used for selection of the conjugants. The obtained clones were then confirmed by PCR using primers flanking the mutant allele (BPSS1385-F1 and BPSS1385-R2).

After that, the positive conjugants were streaked onto yeast extract tryptone (YT) agar (Yeast Extract & Tryptone, BD; Agar, Oxoid) containing 15% (*w/v*) sucrose and 50 µg/mL X-Gluc (YT-sucrose-X-Gluc plates), and incubated at 25 °C for 72 hrs. The resultant colonies were purified by re-streaking on YT-sucrose-X-Gluc plates. For complementation, the same pEXKm5-based allele exchange approach was utilized. The PCR amplicon (1639 bp) containing wild-type *B. pseudomallei cif* sequence was generated by BPSS1385-F1 and BPSS1385-R2 primers. Similarly, the full-length of *cif* was cloned into pEXKm5 and transformed into *E. coli* RHO3 for conjugation with the *B. pseudomallei cif*-deleted mutant. Sucrose selection was employed for merodiploid resolution, resulting in the generation of wild-type sequences and strains that maintained the deletion alleles.

The result of deletion and complementation of *cif* was validated using PCR and immunoblotting. Amplification was carried out using the mutant deletion allele flanking primers (BPSS1385-F1 and BPSS1385-R2) and the primers that were designed to target the oriT region, ensuring that the oriT on pEXKm5 plasmid backbone sequences were absent [39]. Furthermore, the successful construction of the *cif*-deleted mutant and the complemented stains were indicated by immunoblotting using antibodies against Cif protein [29]. Whole cell lysates prepared from *B. pseudomallei* strains were extracted and tested as previously described [29].

### 2.4. Invasion and Intracellular Replication Assay

Human SH-SY5Y cells were seeded at a density of 5 × 10^4^ cells per well in a 24-well cell culture plate. The next day, the medium was removed and replaced with 200 µL of fresh antibiotic-free DMEM. Overnight cultures of *B. pseudomallei* strains were adjusted to 1 × 10^6^ cells per ml by OD measurement at 600 nm and used to infect the cells at a multiplicity of infection (MOI) of 1 to 100. After 2-h co-culturing, the infected cells were washed twice with PBS, and then 500 µL of fresh DMEM containing 250 µg/mL kanamycin (Sigma) was added and incubated at 37 °C for 1 h to eliminate any extracellular bacteria. To recover the invading bacteria, *B. pseudomallei*-infected SH-SY5Y cells were then washed thrice with PBS before cell lysis with 100 µL of 0.1% (*w/v*) Triton X-100. The number of viable bacteria was determined as colony forming units (CFUs) by performing a serial dilution. Ten microliters of each dilution were dropped onto LB agar plates and incubated at 37 °C for 24–48 h. At 4, 6, 8, and 10 h post-infection, the intracellular bacteria were recovered as described above to assess intracellular replication of *B. pseudomallei* strains in human SH-SY5Y cells.

### 2.5. Investigation of Actin-Tail Formation

One day before infection, SH-SY5Y cells were plated on 22 × 22 mm square glass coverslips (Menzel-Glaser) in a 6-well plate (Costar, Corning, NY, USA) and incubated at 37 °C in a humidified 5% CO_2_ atmosphere. The overnight *B. pseudomallei* culture was subjected to infection by the SH-SY5Y cells at an MOI of 20. After killing the extracellular bacteria as previously described, actin tail formation was observed at 6 h post-infection. The infected cells were washed with PBS twice and then fixed with 4% (*v/v*) paraformaldehyde in PBS at room temperature overnight. The fixed cells were washed with PBS before permeabilization with 0.5% (*v/v*) Triton X-100 in PBS. After 30 min of incubation, 1% (*w/v*) bovine serum albumin (Sigma-Aldrich, St. Louis, MO, USA) in PBS was added and incubated for 30 min at room temperature. Subsequently, bacteria were stained using a mouse monoclonal anti-*B. pseudomallei* lipopolysaccharide antibody (Camlab, Cambridge, United Kingdom) followed by Alexa Fluor488-conjugated anti-mouse Immunoglobulin (Molecular Probes, Eugene, OR, USA). Actin filaments and DNA were stained using Alexa Fluor555-conjugated phalloidin (Molecular Probes) and 4′,6′diamidine-2′-phenylindole dihydrochloride (DAPI; Molecular Probes), respectively. Actin-tail formation was examined in 100 fields by confocal laser scanning microscopy (LSM 700; Carl Zeiss, Jena, Germany).

### 2.6. Determination of MNGC Formation

At 10 h post-infection, SH-SY5Y cells infected with *B. pseudomallei* strains were stained with Giemsa (Merck, Darmstadt, Germany) as described previously [40]. MNGC formation, which was defined by at least three nuclei in a cell, was evaluated in 100 fields of view using an Olympus BX41 microscope. The percentage of MNGC formation was determined using the following formula: (number of nuclei in a multinucleated giant cell/total number of nuclei counted) × 100. A minimum of 1000 nuclei were counted per experiment.

### 2.7. Statistical Analysis

All assays were conducted in triplicate, and an unpaired t-test of three independent experiments was performed using the GraphPad Prism 8 program (STATCON). Results were considered significant at a *p* value ≤ 0.05.

## 3. Results

### 3.1. Optimal B. pseudomallei MOI for Human Neuronal Cell Infection

We observed *B. pseudomallei* in human neuronal SH-SY5Y cells using three clinical isolates (K96243, 576a, and 1530). We also generated *B. pseudomallei* K96243 *cif*-deletion and complemented mutants using a pEXKm5-based allele replacement system (Figure S1). The presence of the *cif* gene and Cif protein were validated by PCR and immunoblotting, respectively. A *cif* was confirmed to be absent from the *B. pseudomallei* K96243 *cif*-deleted mutant and the presence of *cif* in the complemented strain was as expected (Figure S2). To characterize and compare the *B. pseudomallei* strains in human neurons, in vitro growth curves of these strains in culture medium were analyzed. All strains showed the same rate of growth, indicating that the degree of fitness was similar among these strains (Figure S3).

**Figure 1:**
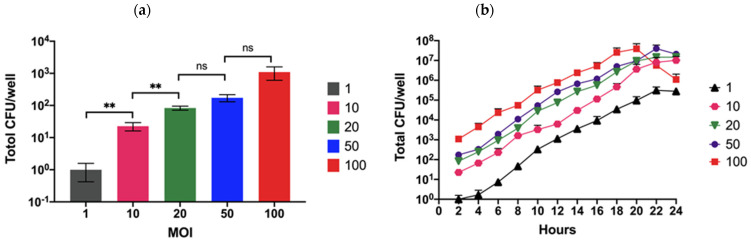
Effects of MOI on invasion and replication of *B. pseudomallei* K96243 in SH-SY5Y cells. SH-SY5Y cells were infected with *B. pseudomallei* K96243 at an MOI of 1, 10, 20, 50, and 100. The numbers of intracellular bacteria were determined by lysing the cells and counting viable bacteria on culture plates. Values are shown as the mean ± SEM of three independent experiments. ns: not significant, ** *p* < 0.01. (**a**) Total numbers of bacteria recovered from SH-SY5Y cells infected with *B. pseudomallei* K96243 at 2 h post-infection. (**b**) Intracellular replication of *B. pseudomallei* K96243 in SH-SY5Y cells at different time points.

The understanding of *B. pseudomallei* pathogenesis in neuronal cells is poor, although data from patient cases with neurological symptoms have accumulated. Here, we used the human neuronal SH-SY5Y cell line to investigate the process of *B. pseudomallei* infection in vitro. The effect of bacterial concentration on cell invasion was determined by co-culturing the reference K96243 strain at a multiplicity of infections (MOI) of 0.1, 0.01, 1, 10, 20, 50, and 100. At a very low MOI of 0.1 and 0.01, the number of bacteria that had invaded cells at 2 h post-infection could not be detected, although internalized bacteria could be recovered 6–8 h after infection (Figure S4). The number of invading bacteria increased with higher MOI, and the number of internalized bacteria significantly increased when using an MOI of 20 (Figure 1a).

However, there was no significant difference between MOI of 20 and 50 and 50 and 100. Moreover, the replication rate of *B. pseudomallei* in SH-SY5Y cells was similar regardless of the number of bacteria added (Figure 1b). It was notable that the number of intracellular bacteria decreased 20 h post-infection when using an MOI of 100. A possible reason for this was that the cells became damaged and released the intracellular bacteria that were then killed in the cell culture medium containing the antibiotic. Moreover, the infected cells may detach from the cell culture plate, leading to a lower number of bacteria recovered from the infected cells. We chose the MOI that gave the maximum number of recovered intracellular bacteria at 2 h post-infection. As a result, an MOI of 20 was chosen for subsequent investigations.

### 3.2. Invasion of Human Neuronal Cells by B. pseudomallei Strains

**Figure 2:**
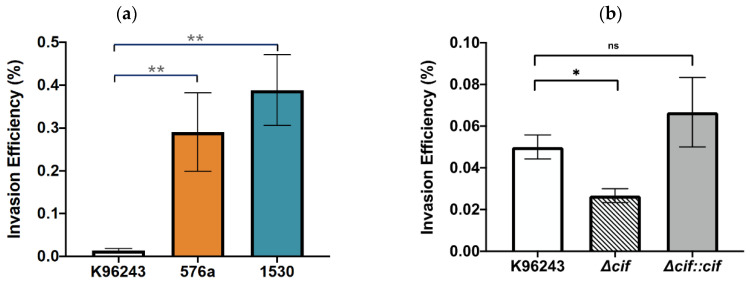
Invasion efficiency of *B. pseudomallei* strains into SH-SY5Y cells. *B. pseudomallei* strains infected SH-SY5Y cells at an MOI of 20. At 2 h post-infection, the invading bacteria were harvested and plated on culture plates for enumeration. Values are shown as the mean ± SEM of three independent experiments. ns: not significant, * *p* < 0.05, ** *p* < 0.01. (**a**) Invasion of *B. pseudomallei* K96243, 576a, and 1530. (**b**) Invasion of *B. pseudomallei* K96243, *cif*-deleted mutant (Δ*cif*), and complemented *cif* mutant strain (Δ*cif::cif*).

The ability of *B. pseudomallei* strains to invade human neuronal cells was examined at 3 h post-infection by comparing the percentage of intracellular bacteria relative to the number of bacteria added to the cells. The percentage invasion efficiency of *B. pseudomallei* reference strain K96243 was 0.014 ± 0.005%, which was significantly lower than those of strains 576a and 1530, at 0.291 ± 0.092% and 0.388±0.082%, respectively (Figure 2a). A contribution of Cif to the invasion of neuronal cells was observed. As shown in Figure 2b, deletion of the *cif* gene caused a significant reduction in invasion efficiency (0.027 ± 0.003%) compared with the wild-type (0.050 ± 0.006%), and complementation of the *cif* mutation fully restored the ability of the *cif*-deleted mutant to invade neuronal cells (0.067 ± 0.017%). These results indicate that Cif is an important factor in the *B. pseudomallei* invasion of neuronal cells.

### 3.3. Intracellular Replication of B. pseudomallei in Human Neuronal Cells

**Figure 3:**
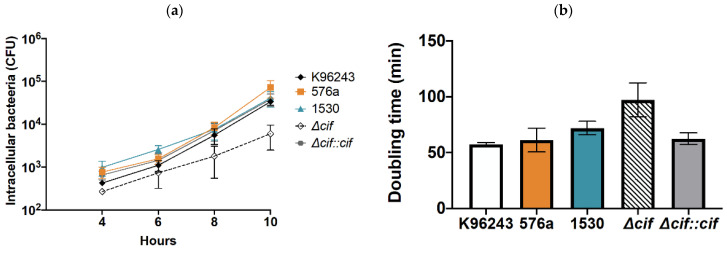
Intracellular survival and replication rate of *B. pseudomallei* strains in SH-SY5Y cells. (**a**) Intracellular replication and (**b**) doubling time were determined for each tested bacterial strain. Data are the mean ± SEM.

After invasion, we tested the ability of *B. pseudomallei* to multiply in SH-SY5Y cells. Intracellular bacteria were recovered by plating on culture medium plates at 4, 6, 8, and 10 hours post-infection. This clearly showed that the number of intracellular bacteria of all the clinical strains increased continuously over time (Figure 3a). We found that the *cif*-deleted mutant survived over time but to a lesser extent than the other strains (Figure 3a). This could result from a lower number of *cif-*deleted mutants that were initially internalized into cells. The growth rates of bacteria were calculated and showed that all *B. pseudomallei* strains replicated similarly in SH-SY5Y cells, with an average doubling time of 68 ± 6 min (Figure 3b). Although the doubling time of the *cif* mutant was approximately 97 ± 15 min, there was no significant difference among strains. This indicates that Cif might not be involved in *B. pseudomallei* intracellular replication.

### 3.4. B. pseudomallei Can Induce Actin-Tail and MNGC Formation in Human Neuronal Cells

**Figure 4:**
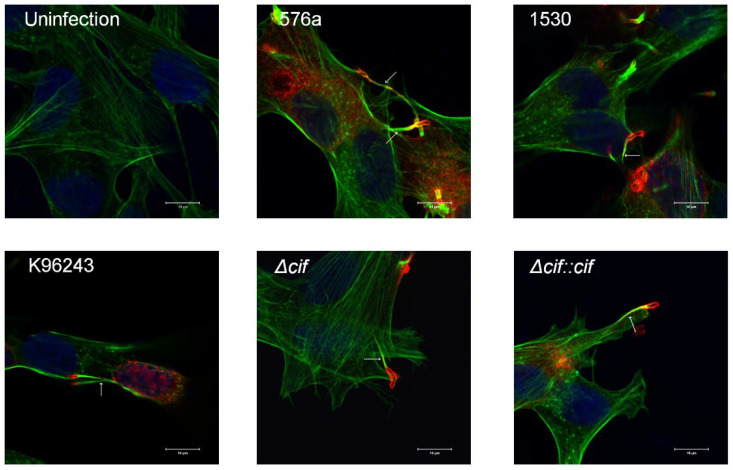
Actin-tails of *B. pseudomallei* strains in SH-SY5Y cells. SH-SY5Y cells infected with *B. pseudomallei* K96243, 576a, 1530, *cif*-deletion, and *cif* complemented strains. At 6 h post-infection, the infected cells were stained to detect actin-tails. Actin-tails in SH-SY5Y cells were examined by direct immunofluorescence staining with Alexa Fluor555-conjugated phalloidin (red) and DNA was stained using Hoechst 33258 (blue). Bacteria were stained using mouse monoclonal anti-*B. pseudomallei* lipopolysaccharide antibody and detected with Alexa Fluor488-conjugated phalloidin (green). Scale bar = 10 µm.

**Figure 5:**
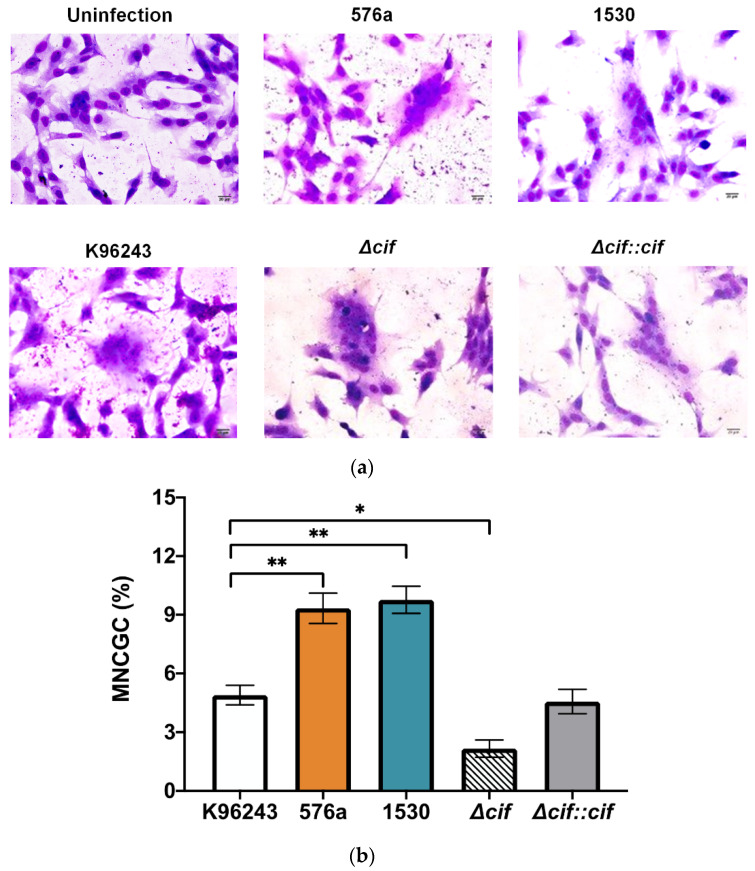
MNGC formation in SH-SY5Y cells. (**a**) MNGC formation induced by *B. pseudomallei* K96243, 576a, 1530, the *cif-*deleted mutant (Δ*cif*), and the complemented *cif* mutant strain (Δ*cif::cif*). Cells were stained with Giemsa. Images were captured by standard light microscopy with a 40× objective lens. (**b**) Percentage of MNGC formation induced by *B. pseudomallei* K96243, 576a, 1530, Δ*cif*, and Δ*cif::cif* strains. Values are shown as the mean ± SEM of three independent experiments. * *p* < 0.05, ** *p* < 0.01.

*B. pseudomallei* is recognized by its ability to induce actin rearrangement that is initiated at one pole of the bacterium tail for intra- and inter-cellular movement. We, therefore, observed actin-tail formation by *B. pseudomallei* strains at 6 h post-infection by confocal analysis. All strains formed actin tails in the neuronal cells with a typical comet-tail phenotype (Figure 4). *B. pseudomallei* exploits actin-based motility for intra- and inter-cellular movement, leading to cell fusion for intracellular survival without exposure to antimicrobial agents or antibodies outside the cells. We detected MNGC formation induced by *B. pseudomallei* at 8 h post-infection (Figure 5a). At 10 h post-infection, we quantified the number of MNGCs and found that the percentages of MNGCs formed by *B. pseudomallei* 576a and 1530 were significantly greater than that of the reference K96243 strain (Figure 5b).

Compared with the wild-type strain, the *cif*-deleted mutant caused similar defective MNGC formation in SH-SY5Y cells (Figure 5b). However, this could be the effect of a lower number of bacteria that survived in the cells at this time (Figure S5). It was possible that the number of MNGCs induced by bacteria lacking *cif* corresponded with the number of intracellular bacteria in SH-SY5Y cells.

### 3.5. Plaque Formation in Human Neuronal Cells

**Figure 6:**
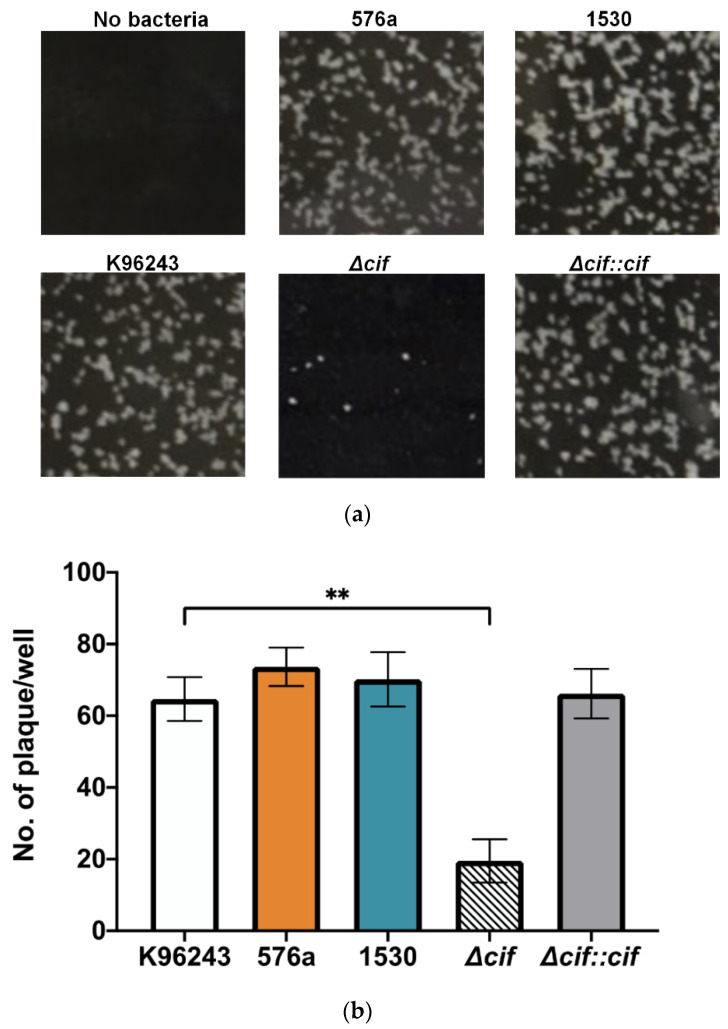
Plaque formation in SH-SY5Y cells. SH-SY5Y monolayer cells were infected with different *B. pseudomallei* strains using an MOI of 20 and incubated for 22 h. (**a**) Representative image of plaque formation in SH-SY5Y cells infected by different *B. pseudomallei* strains (**b**) Plaque-forming efficiency in SH-SY5Y monolayers 22 h after infection. Values are shown as the mean ± SEM of three independent experiments. ** *p* < 0.01.

Following cell fusion, *Burkholderia* spp. spread to adjacent cells, forming plaques as clear zones representing MNGC death [41]. We have shown that all *B. pseudomallei* strains in this study induced MNGCs in human neuronal cells to varying degrees. To assess the virulence of the *B. pseudomallei* strains in SH-SY5Y cells, we determined the number of plaques formed at 22 h post-infection as the last stage of the in vitro infection. Plaque formation induced by K96243, 576a, and 1530 strains was indistinguishable from each other (Figure 6a). Only the *cif-*deleted mutant of *B. pseudomallei* K96243 showed a defect in plaque formation (Figure 6a).

The number of plaques formed by each strain was assessed and is shown in Figure 6B. There was no significant difference between the number of plaques formed by the wild-type strains; K96243 was 65 ± 6, 576a was 74 ± 5, and 1530 was 70 ± 8. The number of plaques formed by the *cif*-deleted mutant was, however, significantly reduced at 20 ± 6. This defective phenotype was recovered when complemented with the *cif* gene; the number of plaques formed by the complemented strain was 66 ± 7. Similar to the number of MNGCs formed by the *cif-*deleted mutant, a decreased number of plaques could reflect the lower number of *cif*-deleted mutant bacteria in cells at 10 h post-infection (Figure S5). This indicated that Cif might not play a role in *B. pseudomallei* dissemination among human neuronal cells.

## 4. Discussion

Upon infection of a human host, *B. pseudomallei* can invade many systems, including the central and peripheral nervous systems [9,42]. Previous studies in mice show a pathway for *B. pseudomallei* into the CNS, the brain stem, and spinal cord via the olfactory and trigeminal nerves [43,44]. However, the underlying mechanism of *B. pseudomallei* pathogenesis and neuronal infection remains poorly understood. In the present study, we examined the *B. pseudomallei* pathogenic process in human neuronal cells. Based on co-culture assays with different *B. pseudomallei* strains and the immortalized bone marrow-derived SH-SY5Y neuroblastoma cell line, the result showed that all tested *B. pseudomallei* wild-type strains harbor the bacterial abilities of invasion, intracellular replication, and formation of actin-tails, MNGCs, and plaques.

Few studies have used neuronal cells to investigate the pathogenesis of bacterial pathogens. *Borrelia burgdorferi*, a bacterium that causes Lyme disease, induces apoptosis of SH-SY5Y cells when surrounded by microglia, the resident macrophage cells of the CNS [45]. However, *B. burgdorferi* is not toxic to neurons; instead, surrounding inflammation produced by microglia in response to *B. burgdorferi* causes the SH-SY5Y cells to die [45]. For *B. pseudomallei*, primary neuron cells have been used in in vitro assays [46], and *B. pseudomallei* MSHR520, a clinical isolate from an Australian patient, can infect glial cells isolated from the olfactory and trigeminal nerves [46].

Several *B. pseudomallei* virulence factors facilitate successful infection. Here, we focused on the Bsa T3SS virulence factor, Cif. We used a *B. pseudomallei cif-*deleted mutant that was defective in the *cif* gene to investigate *B. pseudomallei* infection of human neurons. Although insertional mutagenesis is a popular approach to mutate or disrupt a target gene, a cassette or residual scar is left behind that might affect the transcription of downstream genes (polar effect). Therefore, the *cif-*deleted mutant was constructed by a gene deletion technique.

Based on invasion efficiency, Cif appears to play an important role in *B. pseudomallei* invasion. The *cif-*deleted mutant had reduced ability to invade SH-SY5Y cells, consistent with the role of Bsa T3SS in the penetration of *B. pseudomallei* in other cell types, including epithelial cells, skin fibroblasts, and macrophages [16,40,47]. A *bopE* mutant of *B. pseudomallei* (defective in producing the BopE effector of Bsa T3SS) impairs entry into HeLa cells [48]. Moreover, a *B. pseudomallei bsaQ* mutant (defective in producing a structural component of Bsa T3SS) demonstrates reduced ability in invasion into non-phagocytic cells [49]. In addition to *B. pseudomallei*, T3SS is required for invasion by many other bacteria, such as *Salmonella* spp. and *Shigella* spp. [50]. Although the role of Cif in bacterial invasion remains unclear, our finding could be related to Cif interfering with host cell functions. Like other pathogenic bacteria, *B. pseudomallei* have developed sophisticated arsenals of virulence factors that hijack the host ubiquitin to their own benefit during infection [51]. Previous studies discovered that Cif, specifically deamidated ubiquitin-like protein Nedd8, resulted in the inhibition of CRL, leading to the induction of cell cycle arrest [31,32,33], but not only host cell cycle arrest. Protein ubiquitination also plays crucial roles in controlling eukaryotic cell homeostasis and pathogenesis of neoplastic, infectious, and neurodegenerative diseases [51]. *B. pseudomallei* Cif has been shown to modify host central signaling pathways by activating MAPK/ERK signaling to induce the phosphorylation of the pro-apoptotic protein Bim (Bcl-2 interacting mediator of cell death), and potentially leading to a proapototic effect [34]. Additionally, Cif was shown to increase basal NF-κB activity and stimulate the intracellular replication of the *B. thailandensis* [35]. Thus, it is possible that *B. pseudomallei* Cif is delivered directly into host cells through a Bsa T3SS where it modulates host cell function that promotes bacterial invasion. On the other hand, Cif expression may function coordinately with other bacterial effectors to promote its invasion. Further investigation is needed to clarify these points.

*B. pseudomallei* is able to replicate and survive inside mammalian host cells [52]. During survival inside the host cell, *B. pseudomallei* Bsa T3SS is triggered to deliver effector proteins [53]. *B. pseudomallei* Bsa T3SS is vital for *B. pseudomallei* to evade the endocytic vesicle and survive in the cytoplasm of J774.2 murine macrophage-like cells [54]. We previously found that a *cif* mutation did not affect the capability of *B. pseudomallei* to survive in skin fibroblasts [40]. Similarly, we found that a *B. pseudomallei cif-*deleted mutant did not exhibit defective intracellular replication in human neuronal cells. These results contradict a previous study that showed the direct involvement of *B. pseudomallei* Cif in *B. thailandensis* intracellular replication in HEK293T cells—although the effect was modest—but not on invasion [35]. A possible explanation for this could be that Cif is not present in *B. thailandensis* and the level of expression of *B. pseudomallei* Cif by transfection of Cif plasmid in HEK293T cells might affect the proliferation of *B. thailandensis.*

This study demonstrated that *B. pseudomallei* induces MNGC formation in a human neuronal cell line to different degrees depending on the strains. MNGC formation is also dependent on the cell type infected. For example, MNGC formation by *B. pseudomallei* strain MSHR520 in Schwann cells of trigeminal nerves is greater than that in olfactory ensheathing cells [46]. The *B. pseudomallei cif*-deleted mutant showed reduced MNGC formation in neuronal cells, despite demonstrating efficient actin tail formation comparable to that of the wild-type strain. This phenotype was consistent with the small number of plaques formed by *B. pseudomallei*-infected neuron cells. This finding was also consistent with the lower number of intracellular bacteria in this cell type. It is likely that induction of MNGC in SH-SY5Y cells is linked to the ability of intracellular bacterial replication and plaque formation. However, MNGC formation in RAW 264.7 cells, a murine macrophage-like cell line, by *B. pseudomallei* MSHR520 is not connected to the capacity for intracellular replication [55]. Defective plaque formation of T6SS-1 mutants was observed, though these mutants were able to replicate with a high number of intracellular growth [41]. Thus, the role of *B. pseudomallei* Cif in MNGC and plaque formation in neuronal cells requires further investigation.

To date, only intracellular motility A (BimA) has been shown to play an essential role in CNS invasion and infection [44]. Additionally, an allele of *Burkholderia* BimA significantly impacts the clinical presentation and outcome of patients with CNS melioidosis [56]. *B. pseudomallei* MSHR668 is a clinical isolate with documented central nervous system infection and harbors the *B. mallei*-like *bimA* allele that is believed to be associated with the development and severity of CNS melioidosis [57,58]. However, other virulence factors that contribute to the complications of CNS melioidosis need to be explored. For example, lipopolysaccharide is likely to be associated with pathogenicity and clinical presentation in humans [59]. *B. pseudomallei* K96243 displays type A lipopolysaccharide, while *B. pseudomallei* 576a displays type B [60].

## 5. Conclusions

We showed that *B. pseudomallei* can invade human neuronal cells, multiply, form actin tails, and induce MNGCs. Cif is a virulence factor of *B. pseudomallei* during the process of neuronal invasion. These findings provide new insight into *B. pseudomallei* infection of neuronal cells that will inform the prevention and treatment of neurological melioidosis.

## Figures and Tables

**Table 1:** Primers used in this study.

Primer Name	Sequence (5′-3′)	Purpose	Size (bp)	Source
BPSS1385 F1	CATGTGCGATCATGCAATTT	Upstream BPSS1385	304	This study
BPSS1385 R1	GCGGGCTACTTGGGAGTT	Upstream BPSS1385		
BPSS1385 F2	AACTCCCAAGTAGCCCGCTAGCGAAACCACGAAGAGGT	Downstream BPSS1385	283	
BPSS1385 R2	CTACGGCCACGACCAAGAT	Downstream BPSS1385		
BPSS1385 F	AGAGGCTGCTAATCCACCC	Full length BPSS1385	1053	
BPSS1385 R	ACATCTGCTGCGGTCTCAC	Full length BPSS1385		
OriT-F	TCCGCTGCATAACCCTGCTTC	Validation of the presence of pEXKm5 plasmid backbone	236	[39]
OriT-R	CAGCCTCGCAGAGCAGGATTC			

## Data Availability

The data presented in this study are available on request from the corresponding author.

## Supplementary Materials

The following supporting information can be downloaded at: https://www.mdpi.com/article/10.3390/biology11101439/s1, Figure S1: Construction of *B. pseudomallei cif*-deleted mutant and a complemented strain; Figure S2: PCR -Western Blot; Figure S3: The growth curve in LB medium culture of *B. pseudomallei* strains used in this study; Figure S4: Intracellular replication of *B. pseudomallei* K96243 in SH-SY5Y cells at MOI of 0.1 and 0.01; Figure S5: The number of intracellular *B. pseudomallei* at 10 h post-infection in the SH-SY5Y cells.
