# Supplementary material for: Cycle-Inhibiting Factor Is Associated with Burkholderia pseudomallei Invasion in Human Neuronal Cells
Source: Biology (Basel). 2022 Oct 1;11(10):1439. doi: 10.3390/biology11101439 (PMC9598235; doi:10.3390/biology11101439)
Supplement: Supplementary file 1 [file biology-11-01439-s001.zip › biology-1885012-supplementary.pdf]

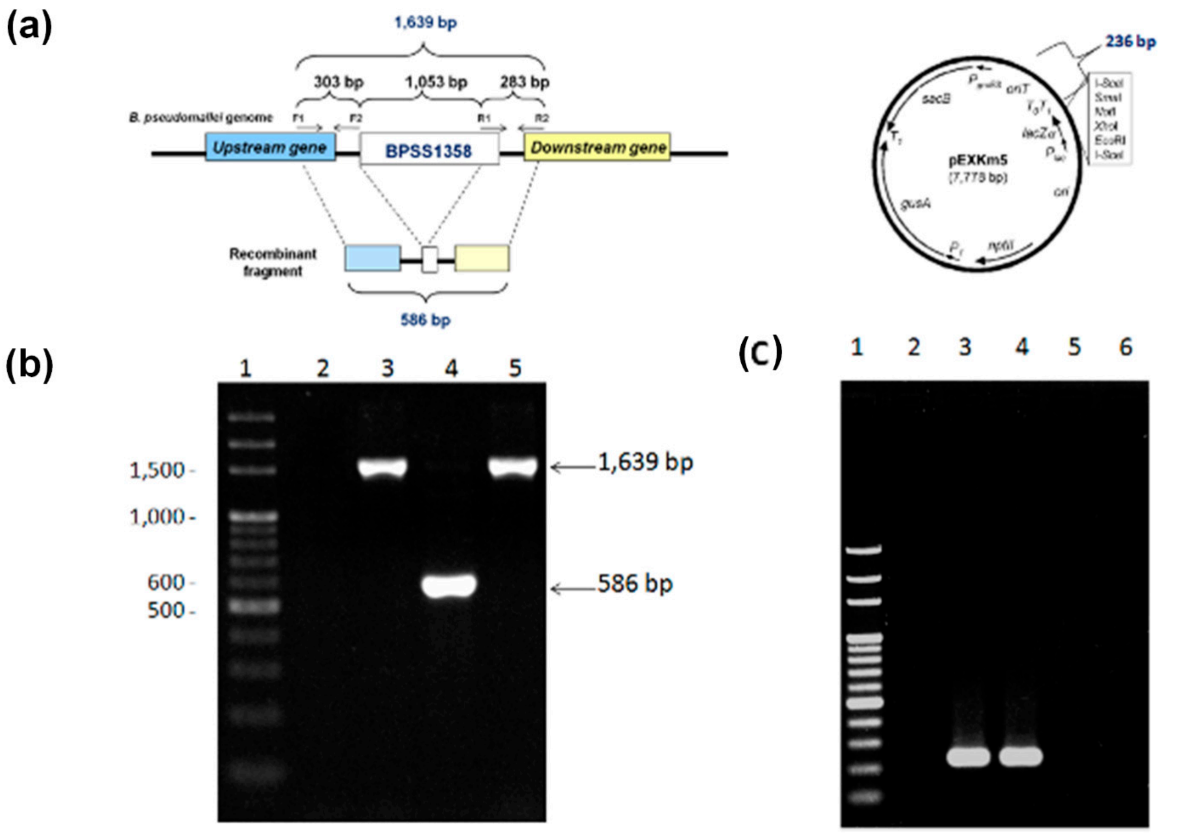

Figure S1

Construction of *cif* deleted mutant and complemented strains. (a) Mutation PCR analysis using primers flanking deleted alleles confirmed the deletion of the *cif* gene on the *B. pseudomallei* chromosome. (b) As expected, a 586 bp DNA fragment (lane 4) was detected in *cif* deleted mutant, whereas a 1,639 bp DNA fragment (lane 3) was detected in the wild type K96243, indicating homologous recombination by deletion of 1,053 bp of *cif* gene on the chromosome of *B. pseudomallei* mutant. The *cif* complement was constructed using the same strategy. The amplified product of the complemented strain showed the presence of a 1,639 bp DNA fragment (lane 5), as observed in the parental strain. (c) PCR analysis of a 236 bp oriT fragment demonstrated an extinction of the pEXKm5 plasmid backbone in the mutant and complement strains (lanes 3 and 4, respectively). The pEXKm5 plasmid was removed from the *cif* deleted mutant, and the *cif* deleted mutant strains were complemented by sucrose selection. The absence of a 236 bp oriT amplicon indicated the removal of pEXKm5 plasmid from the chromosome of *B. pseudomallei cif* deleted mutant and complemented strains (lanes 5 and 6, respectively).

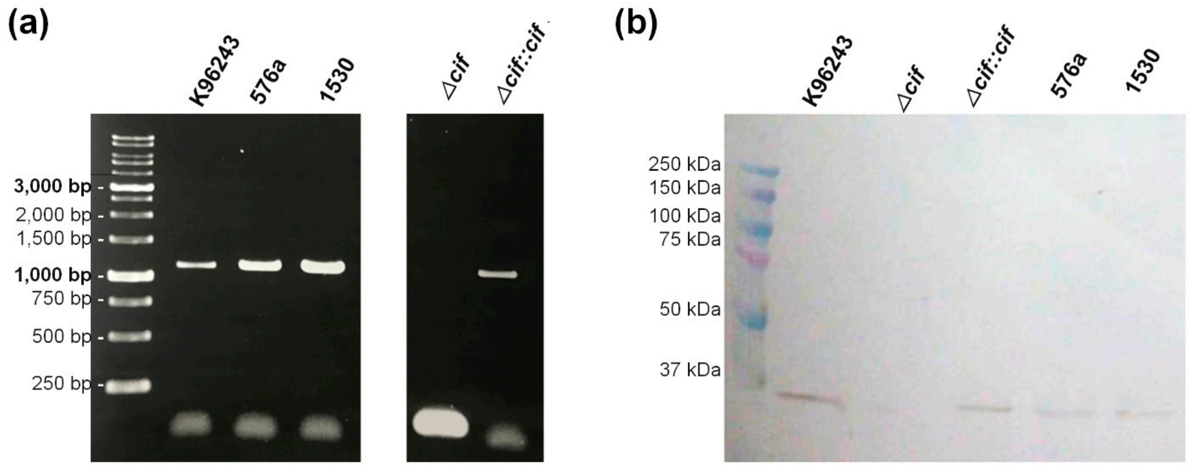

Figure S2

PCR -Western Blot. (a) Amplification of full length *cif* gene in *B. pseudomallei.* (b) Detection of Cif protein in *B. pseudomallei.*

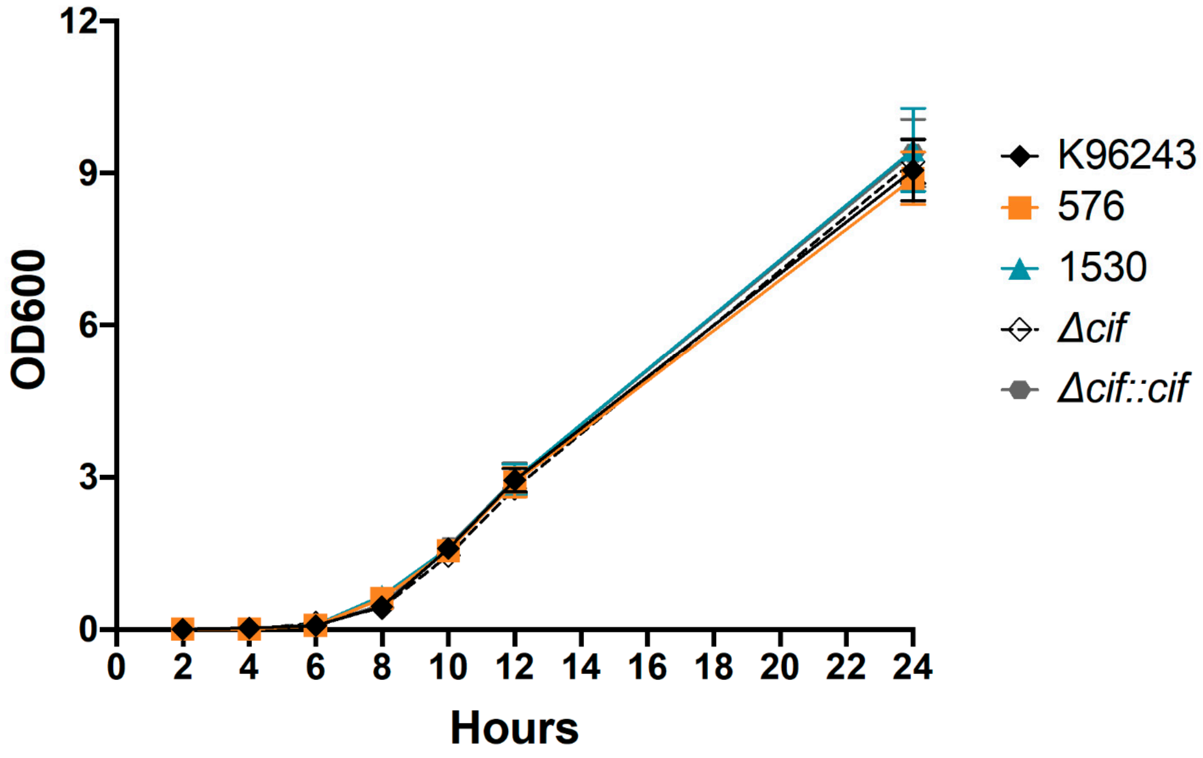

Figure S3

The growth curve in LB medium culture of *B. pseudomallei* strains used in this study was measured by OD reading at 600 nm.

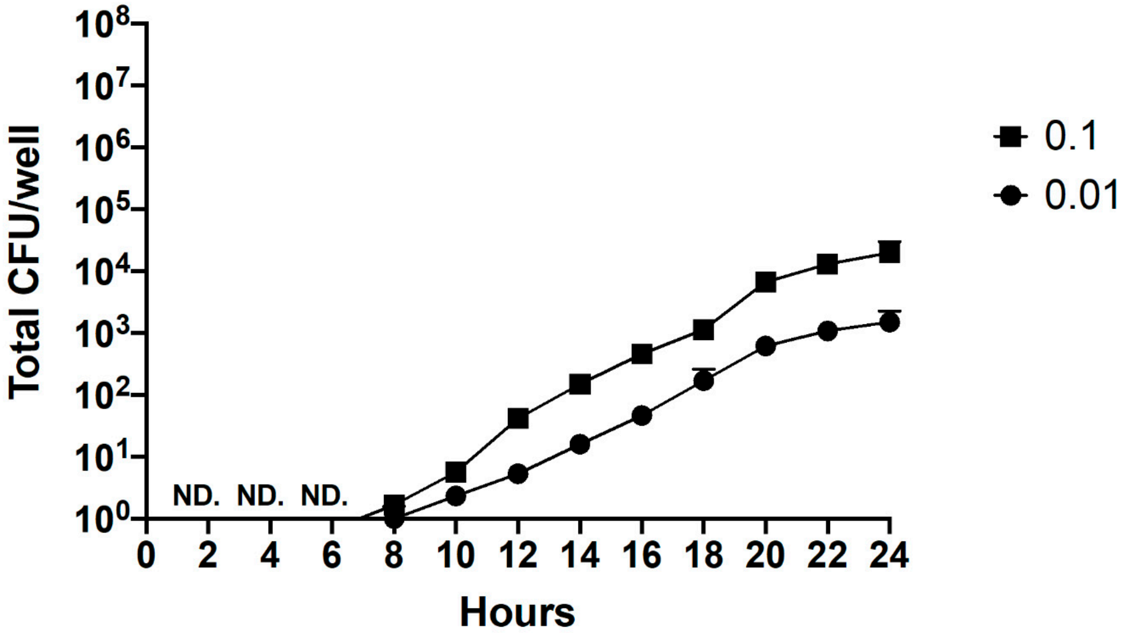

Figure S4

Intracellular replication of *B. pseudomallei* K96243 in SH-SY5Y cells at MOI of 0.1 and 0.01. All assays were performed in triplicate, and error bars represent ±SEM. ND.; Not detectable.

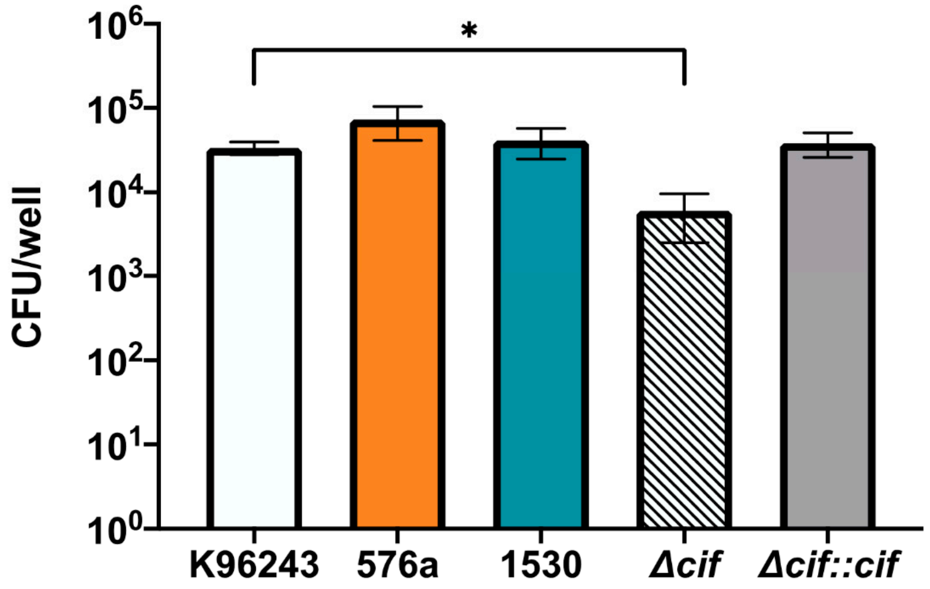

Figure S5

The number of intracellular bacteria at 10 hours post-infection of *B. pseudomallei* strains in the neuronal cells. SH-SY5Y cells were infected with different strains of *B. pseudomallei* at a MOI of 20. The intracellular bacteria were recovered at 10 hours post-infection. Values are shown as the mean ± SEM of three independent experiments. * $p < 0.05$.
